# Poinsettia protoplasts - a simple, robust and efficient system for transient gene expression studies

**DOI:** 10.1186/1746-4811-8-14

**Published:** 2012-05-04

**Authors:** Andrea Pitzschke, Helene Persak

**Affiliations:** 1 Dept. Applied Genetics and Cell Biology, University of Natural Resources and Life Sciences, Muthgasse 18, University of Natural Resources and Life Sciences, 1190, Vienna, Austria

**Keywords:** Protoplast, Transient expression, High transformation efficiency, Chlorophyll autofluorescence, Promoter activity studies

## Abstract

**Background:**

Transient gene expression systems are indispensable tools in molecular biology. Yet, their routine application is limited to few plant species often requiring substantial equipment and facilities. High chloroplast and chlorophyll content may further impede downstream applications of transformed cells from green plant tissue.

**Results:**

Here, we describe a fast and simple technique for the high-yield isolation and efficient transformation (>70%) of mesophyll-derived protoplasts from red leaves of the perennial plant Poinsettia (*Euphorbia pulccherrima*). In this method no particular growth facilities or expensive equipments are needed. Poinsettia protoplasts display an astonishing robustness and can be employed in a variety of commonly-used downstream applications, such as subcellular localisation (multi-colour fluorescence) or promoter activity studies. Due to low abundance of chloroplasts or chromoplasts, problems encountered in other mesophyll-derived protoplast systems (particularly autofluorescence) are alleviated. Furthermore, the transgene expression is detectable within 90 minutes of transformation and lasts for several days.

**Conclusions:**

The simplicity of the isolation and transformation procedure renders Poinsettia protoplasts an attractive system for transient gene expression experiments, including multi-colour fluorescence, subcellular localisation and promoter activity studies. In addition, they offer hitherto unknown possibilities for anthocyan research and industrial applications.

## Background

Transient gene expression is a common approach for studying subcellular localisation, promoter activities or protein complexes in vivo. The most frequently used transient transformation strategies involve: i) *Agrobacterium tumefaciens*-mediated transformation of leaves ii) biolistic approach, i.e. the bombardment of plant tissue (e.g. onion peel) with gold particle-loaded DNA and iii) protoplast transfection. Each method has certain merits and demerits. Leaf infiltration (usually tobacco) with *Agrobacterium* is easy to perform, but many plant species are recalcitrant to this type of transformation. Also, the presence of *Agrobacterium* may alter the activity of several plant proteins. This aspect should be considered while interpreting the data obtained from studies of stress signalling components involving *Agrobacterium*-mediated transformations. For instance, *Agrobacterium* alters the activity of several plant proteins [1,2]. Thus, their function cannot be studied in the native state of the plant, i.e. uncoupled from the pathogen effect. Cell bombardment causes severe tissue damage, requires expensive equipment and frequently yields relatively low transformation rates. The third strategy, protoplast transfection, involves protoplast isolation from plant tissue by enzymatic removal of the cell wall and subsequent transfer of plasmid DNA carrying genes of interest. Transgene expression can usually be observed 16 to 48 hours post transformation. For plant species recalcitrant to *Agrobacterium*-mediated transformation, protoplast transformation and the subsequent attempt to generate entire plants may be a valuable alternative approach for obtaining stable transgenic plant lines.

Genetic transformation of protoplasts has been reported for diverse plant species, including those of e.g. Brassicacea, Solanacea and some ornamental plant families (reviewed in [3]). However, protoplast isolation, transformation as well as downstream analyses are often hampered by a number of factors. For cell culture-derived protoplasts, the plant cell cultures need to be established, which is time-consuming, cost-intensive and requires specific laboratory equipment (e.g. sterile laminar hood, temperature-regulated shaker). In addition, there is a permanent risk of microbial contamination of the cell culture.

These problems are partially circumvented when using mesophyll-derived protoplasts. Many mesophyll protoplast isolation procedures involve the (not necessarily sterile) cutting of leaves, followed by enzymatic lysis of the cell wall and separation of released protoplasts from non-protoplasted tissue debris. However, there is significant tissue damage, often accompanied by a high proportion of broken protoplasts in the final isolates.

In a recent study, Wu et al. [4] reported isolation and transformation of *Arabidopsis thaliana* protoplasts using the so-called “tape-Arabidopsis-sandwich” method. In this, the protoplasts are isolated by pulling leaf layers apart using sticky tapes. The leaf layers attached to the tapes are then exposed to a suspension of cell wall-degrading enzymes. The protoplasts consequently released are harvested by centrifugation. Despite the undeniable break-through of the “tape-Arabidopsis-sandwich” method and its evident advantage for the study of well-characterised ecotypes and mutants, we see a number of technical and particularly biological limitations. We therefore sought an alternative protoplast system that would be complimentary to the Arabidopsis protoplast system and equally simple in its application.

Arabidopsis need to be sown and grown routinely to secure continuous supply of plant material. Since plant growth conditions (light intensity, day/night length and humidity) largely determine protoplast yield and transformation efficiencies [4], the availability of well-controlled climate chambers is a prerequisite.

The major draw-back of protoplasts derived from green leaves is their high content of chloroplasts and chlorophyll, which impedes certain microscopical applications and protein analyses: The strong autofluorescence of chlorophyll can mask the signal of fluorescent-tag-labelled proteins in UV microscopy [5,6]. To some extent, this problem can be alleviated through the use of costly narrow-bandpass filters.

Another chloroplast-associated limitation is the high abundance of photosynthesis-related proteins, particularly ribulose bisphosphate carboxylase (RuBisCo) and light harvesting complex a/b protein (LHC) in mesophyll-derived protoplast protein extracts. In fact, Lhcb1 is the most abundant chlorophyll a/b-binding protein in eukaryotic phototrophs and is often coded by several genes. Due to their abundance, RuBisCo and LCH can impede immuno-detection of proteins of interest due to masking effects or non-specific cross-reactivity with antibodies (reviewed in [7]). Chloroplast-associated complications might be prevented using etiolated leaves. However, transformation efficiencies in Arabidopsis mesophyll protoplasts are much lower in low-light grown plants as compared to high-light-grown plants [4].

Our needs and expectations of an alternative and complementary protoplast isolation and transformation technique were:

· Continuous supply of plant material for protoplast isolation.

· Source plants should have minimal requirements for growth and plant care; no controlled environment chambers should be required.

· Isolation and subsequent transformation of protoplasts should be simple, fast, efficient and reproducible.

· Isolated protoplasts should be robust, minimizing cell damage during transformation and centrifugation steps.

· Protoplasts should contain few or no chloroplasts in order to minimise the interference of chlorophyll autofluorescence in UV microscopy. For some (e.g. immunoblotting) applications, a lack of certain highly abundant protein species, e.g. chloroplastic proteins RuBisCo (small and large subunit) and LHC, potentially masking or causing nonspecific hybridisation signals at app. 55 kDa, 11 kDa and 26 kDa may also be desirable.

Here, we report an alternative versatile system, Poinsettia (*Euphorbia pulccherrima*), for transient expression studies. Poinsettia plants can be grown in the laboratory and special green house space is not required. The isolation and highly efficient transformation of mesophyll protoplasts largely devoid of chloroplasts is described. The method is very time-efficient, as large quantities of vital protoplasts can be obtained within one day. The transformation efficiency is high (>70%); and compared to many other transformation protocols requires only low amounts of plasmid DNA. In addition, this is the first study to describe protoplast isolation and transformation in Poinsettia. Transformed protoplasts are of fundamental value in basic research, e.g. anthocyan synthesis/degradation, as well as for the ornamental industry.

## Results and discussion

### Identifying a suitable plant species as protoplast source

Our first aim in the search for a robust and simple protoplast isolation/transformation system was to identify a suitable plant species. The species should have modest light- and temperature requirements, be perennial, easily propagated and form large leaves. Poinsettia, in our point of view, carries all the above-mentioned desirable characteristics. Moreover, its red leaves are virtually devoid of chloroplasts. Contrary to the general pattern of colouration changes from green to red during plant senescence, Poinsettia leaves are red when young and turn green over time. The plants can easily be propagated by stem cutting, a procedure routinely used in the ornamental industry. Studies were performed on two Poinsettia varieties, Mars Red and Premium Red.

**Figure 1:**
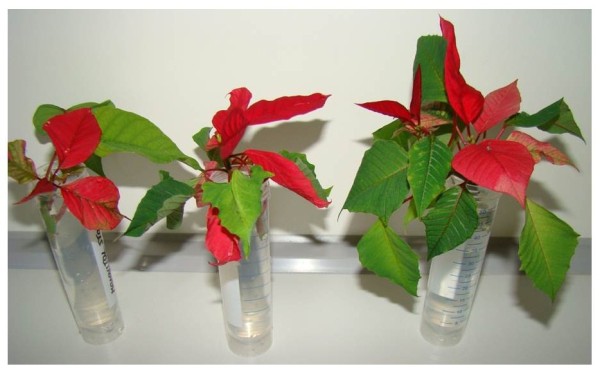
**Stem cuttings of Poinsettia grown in plastic containers filled with tap water.** Red leaves serve as protoplast source.

To test the minimal growth requirements of Poinsettia for our applications, we transferred 7–12 cm long cuttings taken from side branches of a soil-grown “donor plant” to tap water and monitored their appearance over several weeks. The plantlets continued to grow and developed both red and green leaves. Rooting was detectable after 3–4 weeks. Tap-water-grown cuttings exposed to various temperature (20–27°C) and light conditions (16/8 or 8/16 hour regime; green house or window sill, artificial illumination or sun light) appeared as healthy as intact soil-grown donor plants, indicating that Poinsettia has no special growth requirements. Poinsettia produces leaves of 5–15 cm^2^. The young leaves which are red and soft turn green and hard over time. By the time older leaves turn green, the plant has formed fresh red leaves, thus providing a continuous supply of both types of leaves (Figure 1**)**. Together, these properties make Poinsettia a desirable candidate species for protoplast donation.

### Isolation of protoplasts from poinsettia

**Figure 2:**
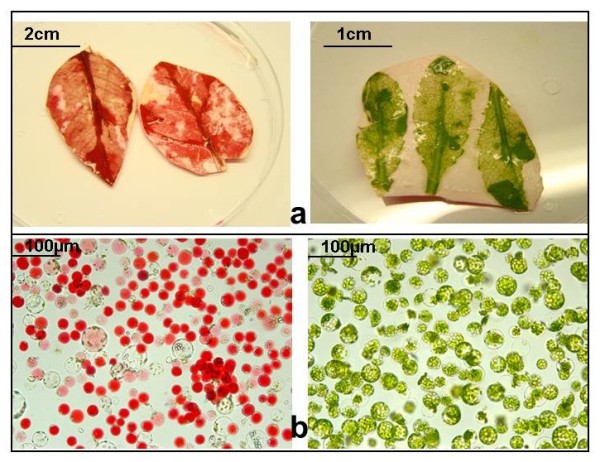
**Isolation of mesophyll protoplasts from red leaves of Poinsettia (left) and Arabidopsis thaliana (right). a)** Lower leaf surface after protoplast release through cellulose digestion. **b)** released protoplasts. Poinsettia protoplasts have large anthocyan-filled vacuoles, whereas Arabidopsis protoplasts contain numerous chloroplasts.

Next, we tested the accessibility of Poinsettia leaves for protoplast isolation by empirically modifying the recently reported “tape-Arabidopsis Sandwich” method [4]. For direct comparison, the procedure was performed with Arabidopsis and Poinsettia in parallel. We found that cell layers of red (i.e. young), but not green (more rigid texture) Poinsettia leaves, can be easily pulled apart. Protoplasts were released from the cell layers through incubation in cellulase/macerozyme solution (Figure 2a). Completeness of cell wall degradation (individual, round cells) was monitored through microscopy (Figure 2b). More than 95% of released cells were viable, as determined by Evans blue staining.

Though protoplasting of Poinsettia leaves takes significantly longer time (overnight) than that of Arabidopsis (20–60 min) [4], Arabidopsis cells are easily “overdigested”, resulting in cell damage and reduced transformation efficiency. Thus it is difficult to optimise the appropriate time point for protoplast harvest. In contrast, Poinsettia protoplasts harvested at 10, 12 or 16 hours of cell wall digestion did not differ in cell viability, completeness of cell wall removal and transformability.

As the starting material, we used red leaves of Poinsettia plants that had been grown at various temperatures and light conditions (see above) and we tested leaves taken from soil-grown plants or from stem-cuttings placed in tap water for 5–30 days. There was no significant difference between these leaf sources, neither with respect to handling during the protoplast isolation procedure nor to the yield, viability or subsequent transformability of isolated protoplasts.

Per gram tissue (app. four medium-sized leaves; 50 cm^2^), 1 to1.5 × 10^8^ protoplasts can be isolated. Poinsettia protoplast yield is thus 3–5 times higher compared to that obtained in the tape-Arabidopsis-sandwich method (3 × 10^7^ cells/g tissue) [4].

### Description of poinsettia protoplast composition, protein profile and pigmentation

**Figure 3:**
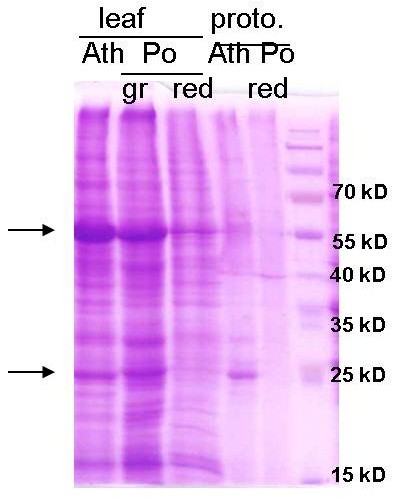
**Protein extracts from Arabidopsis and Poinsettia.** Protein extracts from leaves (10 μg) or protoplasts (5 μg) were separated by 12% SDS-PAGE followed by Coomassie blue staining. Arrows indicate dominant bands at app. 55 and 26 kilo Dalton (kDa), corresponding to the large subunit of RuBisco and LHC, two highly abundant chloroplastic proteins found in samples of green (gr) Poinsettia leaves as well as in Arabidopsis (Ath) leaves and protoplasts, but not in Poinsettia red leaves or protoplasts. No protein extracts of green Poinsettia protoplasts are shown, since the Poinsettia protoplast isolation method is only suitable for red leaves.

Similar to Arabidopsis, Poinsettia protoplast preparations contain cells of various sizes and colouration intensity (Figure 2b). Poinsettia protoplasts isolated from young expanding red leaves are more uniform in size and pigmentation compared to those from expanded leaves. This phenomenon is most likely due to the differently advanced differentiation process of the source tissue. In a small proportion of cells, the vacuole appears to displace other subcellular structures, making it hard to visualize nuclei and cytoplasm. However, in general, the nucleus accounts for approximately 2.5% of the cell area (Additional file 1: Figure S1). We did not observe protoplasts carrying multiple nuclei. Unlike mesophyll-derived protoplasts from other (green) plant species, most Poinsettia protoplasts contain only few chloroplasts (Figure 2b). Accordingly, protein profiles of Arabidopsis and Poinsettia leaves and protoplasts differ substantially: SDS-PAGE/Coomassie blue staining shows absence of dominant protein bands (most likely corresponding to abundant chloroplastic proteins such as RuBisCo and LHC) in red Poinsettia leaves or protoplasts, normally found in Arabidopsis leaves and mesophyll protoplasts and in Poinsettia green leaves (Figure 3).

Red plant pigments, particularly in senescing leaves and ripening fruits, originate mainly from carotinoids, which are synthesised in chromoplasts. We compared the origin of colouration in protoplasts of red pepper (*Capsicum annuum*) fruit and Poinsettia. Cells of red pepper fruit but not of red Poinsettia leaves contain numerous chromoplasts **(**Additional file 2: Figure S2). Apparently, red Poinsettia protoplasts owe their colouration primarily to anthocyans, which are water-soluble compounds that are deposited in the vacuole. These anthocyans can be rapidly extracted using acidified methanol. They show a maximum absorbance at 530 nm (Additional file 3: Figure S3). It is conceivable that for industrial applications or plant pigmentation research, the effect of ectopic gene expression (or certain treatments) on Poinsettia colouration can be conveniently monitored through quantitative and qualitative absorbance scans.

### Transformation of protoplasts

**Figure 4:**
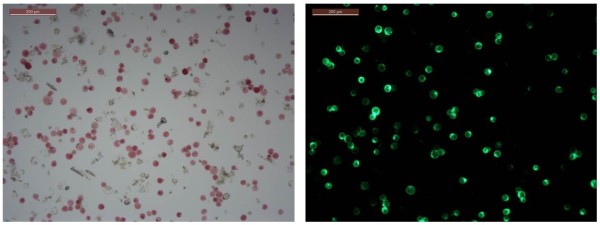
**High-efficiency transformation of Poinsettia protoplasts.** Lower leaf surface after protoplast release through cellulose digestion. 5 × 10^5^ Poinsettia Protoplasts were transformed with 0.7 μg of a DNA construct for constitutive expression of Yellow Fluorescent Protein. YFP expression was visualised after overnight incubation, using a UV microscope. Left: brightfield; right: UV.

**Figure 5:**
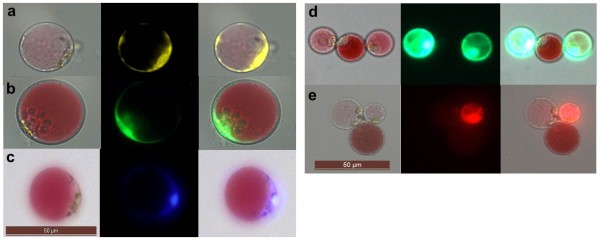
**Multi-colour fluorescence detection in Poinsettia protoplasts.** Poinsettia protoplasts were transformed with DNA constructs for ectopic expression of fluorescent proteins. Left: brightfield, middle: UV image, right: overlay. **a)** VIP1-YFP, **b)** VIP1-GFP, **c)** VIP1-CFP, **d)** YFP, **e)** mCherry. In images **d)** and **e)** also non-transformed protoplast(s) are shown to document the low level of nonspecific/background autofluorescence.

**Figure 6:**
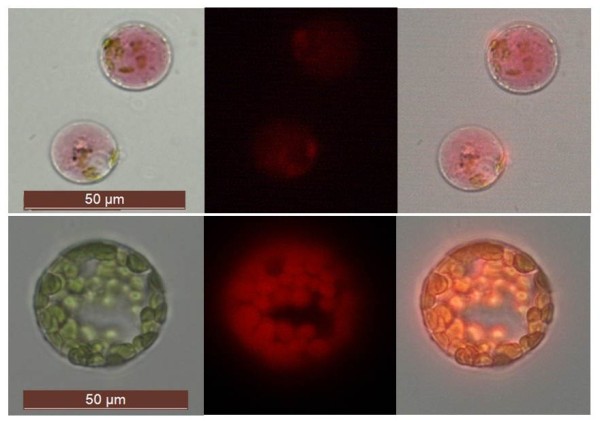
**Autofluorescence in non-transformed Poinsettia vs. Arabidopsis protoplasts.** Excitation with a filter for red fluorescence detection triggers strong autofluorescence in (chloroplast-rich) Arabidopsis protoplasts (bottom row), but not in Poinsettia protoplasts (top row). Interference of chlorophyll autofluorescence with red fluorescent protein detection (e.g. mCherry) can be overcome in Poinsettia (Figure 5e). Left: brightfield,middle: UV image, right: overlay.

a) Optimisation of transformation parametersWe adapted the polyethylene-glycol (PEG)-based technique for Poinsettia protoplast transformation. Volumes of protoplast solution, plasmid DNA amounts, washing procedures and centrifugation conditions were optimised empirically. A dilution series of plasmids (5, 3, 1.5, 0.7, 0.3, 0.15 μg) was used to identify the minimal amount of DNA required for transformation. We used a construct encoding Yellow Fluorescent Protein (YFP), driven by the constitutively active Cauliflower Mosaic Virus (CaMV35S) promoter to monitor transformation efficiency by fluorescence microscopy.PEG-based protoplast transformation protocols include the replacement of PEG by a solution in which the transformed protoplasts are finally incubated. Exchange of the solutions is achieved through repetitive washing steps involving the addition of an appropriate buffer(s) and very gentle (3 times 1 min, 60–100 g) centrifugation. (e.g. [4; 6]). Plant protoplast pellets obtained after such gentle centrifugation are normally fragile, impeding the efficient removal of the supernatant fluid, thus necessitating further washing steps. Centrifugation velocities of 300 g or higher caused protoplast rupture in Arabidopsis protoplasts (not shown). In contrast, Poinsettia protoplasts proved very robust, as a dense pellet of virtually undamaged cells was obtained after centrifugation at 600 g. In fact, even at 1000 g centrifugation, the cells stayed intact (assessed by Evans blue staining, Additional file 4: Figure S4). Thus, the supernatant fluid can be efficiently removed without agitating the protoplast pellet, and further washing step(s) can be omitted. Finally, protoplasts are re-suspended in a standard protoplast solution (W5) and incubated until analysis . The entire transformation procedure takes approximately 20 minutes and can be conveniently performed as one-tube reaction.

b) Transformation efficiencyWe consistently observed high transformation rates (>70%) when using at least 0.7 μg plasmid DNA per 50 μl transformation reaction. Fluorescence in protoplasts transformed with CaMV35S::YFP was diffusely distributed in the cell (Figure 4). Much lower transformation efficiencies were achieved with lower plasmid amounts or non-purified plasmid preparations. Both the plasmid purification kits tested (Qiagen, Promega). were equally suitable. Compared to the previously reported “Tape-Arabidopsis-Sandwich” protocol, in which 0.7 μg per 50,000 cells are used [4], Poinsettia protoplast transformation requires approximately 5 times less DNA (30–40 μg/20,000-100,000 cells) saving costs and time for repetitive plasmid DNA preparations. Comparatively high DNA amounts (10–50 μg/20,000-100,000 cells) are also required for PEG-mediated protoplast transformation of other plant species, e.g. carrot, rapeseed, soybean, tobacco and rice[8-11]. It has been reported that protoplast transformation depends on plasmid size; with smaller plasmids being transformed more easily and requiring less DNA amounts than larger plasmids [12]. The construct tested here, CaMV35S::YFP, is comparatively large (8 kilo base pairs). Whether high transformation efficiencies in Poinsettia can also be obtained with low amounts of even larger-sized (>9 kb) plasmids remains to be determined.

c) Kinetics of expression Provided the promoter is recognised, the transcriptiona l/translational machinery and protein maturation and stability largely determine over which period the post-transformation transgene expression can be detected. Most transformation protocols involve an overnight incubation. Strikingly, fluorescence in YFP-transformed Poinsettia protoplasts was detected as early as 90 min after transformation. When the same construct was transfected into Arabidopsis mesophyll protoplasts, fluorescence was detected only 6 hours post-transformation. Consistently, enzymatic reporter gene activity in Poinsettia protoplasts transformed with a construct driving constitutive expression of ß-glucuronidase (GUS) was observed already within 2 hours post-transformation and further increased later on (Additional file 5: Figure S5). We could still observe YFP fluorescence in Poinsettia protoplasts 4 days after transformation.

d) Multi-colour fluorescence in transformed protoplastsHaving documented ectopic YFP expression, we next assessed the suitability of Poinsettia protoplasts for subcellular localisation/multi-colour fluorescence studies involving a variety of fluorescent protein tags. Protoplasts were transformed with DNA constructs encoding yellow, green, blue, or red fluorescent proteins (VIP1-YFP, VIP1-GFP, VIP1-CFP, free YFP and free mCherry), all driven by the constitutive CaMV35S-promoter. Expression of the three VIP1 fusion proteins in Poinsettia were mainly detected in the nucleus, but also in the cell periphery (Figure 5a-c), consistent with our previous observations in Arabidopsis root cell-culture-derived protoplasts [13]. Fluorescence in Poinsettia protoplasts expressing non-tagged YFP was found distributed throughout the cell (Figure 5d). Importantly, Poinsettia protoplasts display only low levels of background fluorescence. (Figure 5d-e). mCherry-transformed Poinsettia protoplasts exhibited a widely-distributed intensive red fluorescence (Figure 5e), while in Arabidopsis protoplasts mCherry-derived fluorescence remained undetectable. The latter phenomenon is most likely due to the strong chlorophyll-derived autofluorescence, which is masking any additional signal. UV microscopes equipped with narrow band pass filters may overcome this problem. Depending on chloroplast density and the type of microscope, red fluorescence of cytoplasmic proteins in Arabidopsis protoplasts may be detected as very thin rings around the chloroplasts [14,15]. Figures 5e and 6 emphasize the advantage of Poinsettia protoplasts over any green mesophyll-derived protoplasts, particularly for the detection of red fluorescent proteins. Although, anthocyanin compounds are generally classified into fluorescent substances [16], multi-colour fluorescence detection (at least of the four fluorescent tags tested here) did not negatively correlate with the pigmentation intensity.

e) Promoter activity studiesA major advantage of transient expression systems is that comparatively little effort and time required for studying the mutual effect of two or more co-transformed components of interest. Protoplast co-expression systems are widely used to assess e.g. the transactivating/-repressing capacity of transcription factors [17,18] For instance, the reporter gene, ß-glucuronidase (GUS), is fused to a promoter of interest, and the construct is co-transfected with an effector construct (containing a transcription factor or other putative signalling component) [13,19,20].

**Figure 7:**
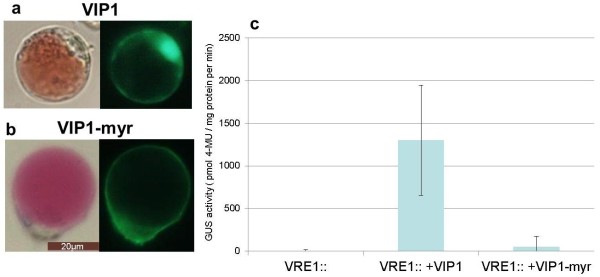
**Quantitative promoter activity studies in Poinsettia protopolasts.** Protoplasts were transfected with a synthetic promoter-GUS reporter gene construct containing the VIP1 response element (VRE1; fused to minimal CaMV35S promoter) alone or in combination with a construct driving overexpression of YFP fusion proteins to **a)** the transcription factor VIP1 or **b)** the membrane-retained VIP1 variant (VIP1-myr), see also figure S6 **c)** After overnight incubation, GUS activity of protoplast extracts was quantified and normalised to total protein amounts. Comparable transformation efficiencies were documented by UV microscopy. Given are mean values and standard deviations of GUS activity (*n* _ 6).

We tested the suitability of Poinsettia protoplasts for quantitative GUS activity assays. For this, protoplasts were transformed with a DNA construct containing the GUS gene driven by the synthetic VIP1 response element (VRE1)-CaMV35S minimal promoter, either alone or with its known activator, the bZIP transcription factor VIP1 [19]. In addition, we compared (VRE1)-CaMV35S minimal promoter induction by VIP1 and VIP1-myr (carrying a myristoylation signal for membrane targeting). Localisation of YFP fusions to VIP1 and VIP1-myr was monitored by UV microscopy. As in Arabidopsis protoplasts [13], a high proportion of VIP1-YFP locates to the nucleus, whereas VIP1-myr is largely retained in the membrane (Figure 7a, b, Additional file 6: Figure S6). Moreover, in line with the dependency of promoter activation on the nuclear localisation of VIP1 in Arabidopsis [13], GUS reporter gene activity is strongly induced by VIP1, but not by VIP1-myr (Figure 7c).

### Additional considerations

Each protoplast system has its limitations. E.g. cellular processes occurring in protoplasts derived from cell cultures or roots are certainly distinct from those in mesophyll cells. Protein profiles of green Poinsettia leaves and Arabidopsis are very similar (Figure 3, lanes 1 + 2 from left). In comparison, red Poinsettia leaf extracts display both similarities and dissimilarities (lanes 1 + 2 vs. lane 3), indicating that the physiology and metabolism of green Poinsettia tissue resembles that of other green plants, but that there are certain differences in red Poinsettia tissue. This difference needs to be considered in certain applications. For instance, cellular responses to treatment with photosynthesis inhibitors or activators may not be studied in the Poinsettia protoplast system.

Protoplasts isolated from young expanding red leaves tend to be more uniform in size and pigmentation (e.g. Figure 2b) compared to protoplasts from expanded leaves (e.g. Figure 4), mirroring the differently advanced differentiation process of the tissue sources. To achieve consistent experimental results, e.g. in treatment-induced reporter gene studies, the use of young leaves (<70% of final size) is advisable. However, a less uniform protoplast source, i.e. fully-expanded leaves, allows localisation studies (including cell responses to treatments) in a mixture of cell types within one experiment, providing more detailed information about protein distribution.

In our studies, we also included two *Euphorbia pulccherima* plants the variety of which was unknown. These plants were equally suitable for protoplast isolation and transformation, suggesting that our method is probably applicable to any Poinsettia variety.

### Summary

As shown in the previous sections, the CaMV35S promoter is functional and drives rapid, strong and durable expression in Poinsettia protoplasts. For the fusion proteins tested in this study, subcellular sorting correlates with observations and/or predictions in Arabidopsis. The expression of yellow, green, blue and red fluorescent fusion proteins can be monitored – rendering the “Poinsettia Protoplast System” suitable for multi-colour-analyses. The early onset of transgene expression compared to Arabidopsis protoplasts might prove useful for studies of proteins/protein complexes that cause cell-death within few hours.

In addition, Poinsettia protoplasts are suitable for effector/reporter gene studies involving GUS activity quantification. The red pigment of the cells does not appear to interfere with ß-glucuronidase activity or its quantification. Relevant applications for GUS activity assays in Poinsettia are e.g. i) to determine promoter activities in a non-Arabidopsis background; ii) to compare responsiveness of a promoter to treatments between plant species; iii) to study promoter activity in a chloroplast-depleted environment. As exemplified by cotransformation of the GUS reporter gene plasmid VRE1-CaMV35S_minimal promoter and its effector VIP1, the system is certainly well-suited for studying synthetic promoter constructs on a large scale (e.g. step-wise mutation analysis).

Due to the low abundance of RuBisCo and LHC, Poinsettia mesophyll protoplasts provide an advantage over mesophyll-derived protoplasts from other species. In addition, the system might be particularly interesting if one desires to assess the interaction/characteristics of candidate proteins in a chloroplast-depleted, i.e. a low-oxygen generating environment.

## Conclusions

Our work reveals the versatility and robustness of Poinsettia (*Euphorbia pulccherrima*) protoplasts as convenient system for transient expression studies. The method is easy to perform and involves a minimum of handling, equipment and costs. It is of potential interest to any plant scientist who has hesitated so far to routinely employ protoplast transient expression systems due to the substantial effort, time and irreproducible transformation efficiencies associated with current protocols. It also offers a valuable alternative to other commonly used protoplast systems, such as Arabidopsis mesophyll or tobacco BY-2 cells. Depending on the particular question of interest, either system can be employed to overcome limitations of the other system.

### Outlook

Further possible fields of application for Poinsettia protoplast include research on anthocyanin synthesis, regulation and metabolism as well as protoplast fusion studies. Poinsettia protoplast expressing a transgene of interest may be fused to (e.g. the less abundant) non-red, chloroplast-containing Poinsettia protoplasts or to mesophyll-derived protoplast from another species. These studies would for instance allow to assess possible effects of the immediate load of a high dose of *in-planta*-produced protein in a naïve cell of interest. The ease of Poinsettia protoplast isolation and transformation is a promising feature for biotechnological applications, but also for the ornamental industry to introduce new characteristics into this intensively-marketed plant.

## Material and methods

### Plant material

Plant growth and protoplast transformation of *Arabidopsis thaliana* Columbia was performed according to [4]. Flowering plants of poinsettia (*Euphorbia pulcherrima* Mars Red and Premium Red) were purchased in a local nursery. For propagation, 10–12 cm cuttings were placed in 50 ml plastic tubes filled with tap water. Plants were grown under ambient temperatures, light and humidity conditions. In general, any healthy-appearing plant is a good source for protoplasts.

### Plasmids for protoplast transformation

The coding regions of the YFP, GFP, CFP or mCherry genes were cloned into a pGreen0029 derivative [21] containing the CaMV35S promoter and nos terminator. The synthetic promoter construct VRE1::GUS, VIP1-YFP and myr-VIP1-YFP have been described previously [13,19]. For VIP1-CFP, the YFP tag of VIP1-YFP was replaced by CFP. *E. coli* plasmid DNA was purified using commercial plasmid preparation kits (Qiagen, Promega). Plasmid purification is essential for achieving high transformation rates.

### Poinsettia protoplast isolation

Only red leaves were used for protoplast isolation. The upper epidermis of leaves was affixed to a strip of tape tissue-glue-tape (X-Way); the lower epidermal surface to Scotch® Magic™ tape (3 M). The tapes were pulled apart, and the tape containing the upper epidermal layer was incubated overnight, at room temperature, on a horizontal shaker (40 rpm) in enzyme solution (1% cellulase, 0,25% macerozyme, 400 mM mannitol, 10 mM CaCl_2_, 20 mM KCl, 0.1% BSA, 20 mM MES pH5.7). (Performing this step in opposite orientation also works, but protoplast yield is approximately 5 times lower.). Protoplasts were transferred to round-bottom 2 ml plastic tubes and pelleted for 3 min at 100 g. The cells were washed twice (2 min, 100 g) with W5 (154 mM NaCl, 125 mM CaCl_2_, 5 mM KCl, 2 mM MES pH5.7) and finally re-suspended in 1 ml W5. After a 30 min incubation on ice, protoplasts were counted, centrifuged (2 min, 100 g) and resuspended in MMg (400 mM mannitol, 15 mM MgCl_2_, 4 mM MES) to a density of 5 × 10^5^ – 1 × 10^6^ cells/ml.

Protoplast isolation from pepper (*Capsicum annuum*) was performed as described above, but using sliced pieces of red fruits instead of taped leaves. Arabidopsis mesophyll protoplast isolation and transformation was performed according to [4].

### Poinsettia protoplast transformation and microscopy

50 μl of Poinsettia protoplasts (5 × 10^5^ – 1 × 10^6^ cells/ml) were pipetted (using cut tips) to 0.7 μg plasmid DNA diluted in 15 μl H_2_O in round-bottom 2 ml plastic tubes. Samples were mixed gently, 150 μl PEG (40% PEG 4000, 100 mM CaCl_2_, 200 mM mannitol) was added. Samples were mixed by ticking against the tube until they appeared homogenous. After 10 min incubation at RT, 1 ml of W5 was added (stepwise, as 2 x 500 μl) and protoplasts were centrifuged at 600 g, 6 min, RT. The supernatant solution was removed, and protoplasts were incubated in 500 μl W5 overnight at room temperature (RT) in the dark. Initially, we observed significant loss of protoplasts after overnight incubation, irrespective of the brand or coating properties of the tube (several suppliers tested; ordinary tubes, special surface-coated tubes, PCR tubes).We found the easiest and most efficient way to prevent protoplast loss due to (electrostatic) plastic adhesion is to add 0.5% BSA to the washing and incubation solutions.

Transformation efficiency was determined through UV microscopy (Leica DM5500B), equipped with excitation/emission filters: BP450-450 nm/LP515 nm; or BP515-560 nm/590 nm (mCherry).

### Cell viability assessment

Evans blue dye was added to the protoplasts in W5 solution to a final concentration of 0.04%. Following a 10 min-incubation at room temperature, viability of protoplasts (freshly prepared or up to 11-d old) was assessed by light microscopy.

### Protein extraction and SDS-PAGE

After overnight incubation, transformed protoplasts were pelleted (30 min, 10,000 g, RT). Integrity of centrifuged protoplasts was confirmed through microscopy. The supernatant fluid was removed, cell pellets were snip-frozen in liquid nitrogen and resuspended in 30 μl protein extraction buffer (25 mM Tris pH 7.7, 15 mM EGTA, protease inhibitor cocktail (Roche)). Protein contents of extracts obtained by centrifugation (14,000 rpm, 10 min, 4°C) were quantified (Bradford assay; Biorad), samples were denatured through addition of 1 volume of 2 × SDS loading buffer and incubation at 95°C, 3 min. Leaf (10 μg) and protoplast (5 μg) proteins were separated by 12% polyacrylamide gel electrophoresis and visualised by Coomassie blue staining.

### Promoter activity studies

Poinsettia protoplasts were transformed with VRE1_35min::GUS alone or in combination with VIP1-YFP or myr-VIP1-YFP. After overnight incubation, cell pellets were snap-frozen. Proteins were extracted in 50 μl 50 mM sodium phosphate pH 7/10 mM EDTA. ß-glucuronidase activity of extracts was quantified using MUG (4-methylumbelliferyl-β-D-glucuronide) as substrate. Substrate conversion was measured in 15 min intervals using a Tecan microtiter plate reader (365 nm excitation/455 nm emission, black 96-well plates Corning). Serial dilutions of 4-MU served as reference standard. Protein content of samples was quantified with Bradford reagent (Biorad).

Poinsettia and Arabidopsis mesophyll protoplasts were transfected with 2 μg of a construct for constitutive expression of the glucuronidase (GUS) reporter gene, driven by the CaMV35S promoter. Protoplast samples were collected 1, 2, 3, 4, 5 and 20 hours post-transformation. GUS activity was quantified as described above.

### Anthocyan absorbance scan

Protoplasts were centrifuged (30 s, 10,000 g, RT) and re-suspended in an equal volume of chilled anthocyan extraction solution (AES; 1%HCl in methanol). The supernatant fluid obtained after another centrifugation step was diluted in AES, transferred to a 96-well transparent microtitre plate (Corning) and analysed using the “absorbance scan” tool of a TECAN infinite 200 microplate reader.

### Visualisation of subcellular structures

#### Staining of nuclei

The nucleic acid binding dye Midori Green (Nippon Genetics) was added to the protoplasts in W5 solution to a final concentration of 0.005%. After 3 min incubation, fluorescence was documented by UV microscopy (Leica DM5500; BP450-450 nm/LP515 nm).

#### Plasma membrane staining

Poinsettia protoplasts were transformed with VIP1-myr-YFP. 20 hours post-transformation the protoplasts were treated with the membrane-binding red fluorescent dye FM4-64 (Invitrogen) at a concentration of 4 mM for 1 hour at room temperature. YFP and FM4-64 fluorescence was detected by UV microscopy.

## Abbreviations

LHC: Light harvesting complex; RuBisCo: Ribulose bisphosphate carboxylase; G/C/YFP: Green/cyan/yellow fluorescent protein; GUS: ß-glucuronidase, myr: myristoylation signal peptide; CaMV35S: Cauliflower mosaic virus 35S promoter; VIP1: virE2-interacting protein 1; VRE1: VIP1 response element 1; RT: Room temperature.

## Competing interests

The authors declare no competing interests.

## Authors’contributions

Both contributing authors (AP and HP) have designed and performed the experiments and written the manuscript. All authors read and approved the final manuscript.

## Supplementary Material

Additional file 1 **Figure S1. Visualisation of nuclei in Poinsettia protoplasts.** Protoplasts were incubated with a 1:20,000 dilution of Midori green, a DNA-binding fluorescent agent. 3 min after incubation, nuclear staining was detected by UV microscopy. Left: brightfield,middle: UV image, right: overlay. Click here for file

Additional file 2 **Figure S2. Red plant pigmentation.** Protoplasts isolated from red pepper fruit (*Capsicum annuum*) owe their colour to numerous carotinoid-rich chromoplasts (right). In contrast, pigmentation in red Poinsettia protoplasts is due to anthocyans that are uniformely distributed in the large vacuole (left). Click here for file

Additional file 3 **Figure S3. Absorbance profile of red Poinsettia protoplasts.** Anthocyans were extracted by lysis of Poinsettia protoplasts in acidified methanol. Absorbance was assessed using the “absorbance scan” tool of a Tecan microtitre plate reader. Click here for file

Additional file 4 **Figure S4. Viability of Poinsettia protoplasts after the transformation procedure and overnight incubation**. Poinsettia protoplasts were incubated for 10 min with Evans blue, a dye which penetrates into non-viable cells. The only non-viable cell contained in this image is indicated by an arrow. Click here for file

Additional file 5 **Figure S5. Kinetics of transgene expression in Poinsettia and Arabidopsis protoplasts.**Protoplasts were transfected with 2 μg of a construct for constitutive expression of the glucuronidase (GUS) reporter gene, driven by the CaMV35S promoter. Protoplast samples were collected for GUS activity quantification at the indicated time points post-transformation. Click here for file

Additional file 6 **Figure S6. Subcellular localisation of VIP1-YFP fused to a myristoylation signal peptide**. Top: Two examples of Poinsettia protoplasts expressing VIP1-myristoyl-YFP. Bottom: Plasma membrane colocalisation of VIP1-myr-YFP. Poinsettia protoplasts were transformed with VIP1-myr-YFP and treated with the membrane-binding red fluorescent dye FM4-64. Fotographs were taken after 1 hour. From left to right: brightfield, YFP channel, red channel, overlay. Note that chlorophyll–derived autofluorescence is contributing to the extended fluorescent region in the red-channel-image. Click here for file
