# Supplementary figures and images for: Poinsettia protoplasts - a simple, robust and efficient system for transient gene expression studies
Source: Plant Methods. 2012 May 4;8:14. doi: 10.1186/1746-4811-8-14 (PMC3478982; doi:10.1186/1746-4811-8-14)

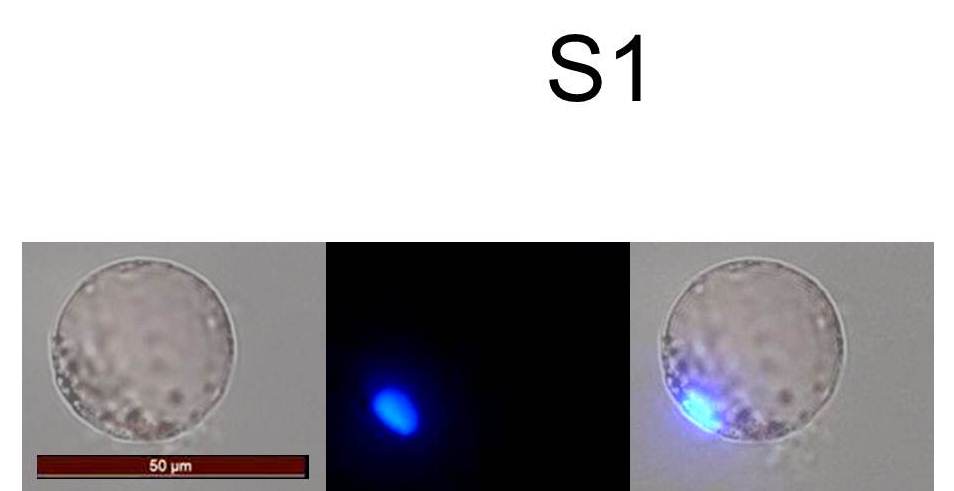

Supplement: Additional file 1 — Figure S1. Visualisation of nuclei in Poinsettia protoplasts. Protoplasts were incubated with a 1:20,000 dilution of Midori green, a DNA-binding fluorescent agent. 3 min after incubation, nuclear staining was detected by UV microscopy. Left: brightfield,middle: UV image, right: overlay. [file 1746-4811-8-14-S1.jpeg]

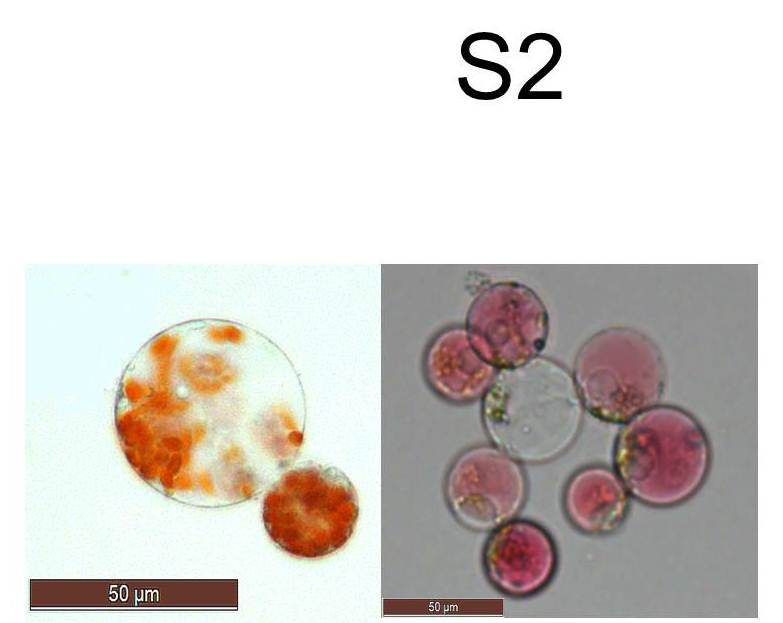

Supplement: Additional file 2 — Figure S2. Red plant pigmentation. Protoplasts isolated from red pepper fruit (Capsicum annuum) owe their colour to numerous carotinoid-rich chromoplasts (right). In contrast, pigmentation in red Poinsettia protoplasts is due to anthocyans that are uniformely distributed in the large vacuole (left). [file 1746-4811-8-14-S2.jpeg]

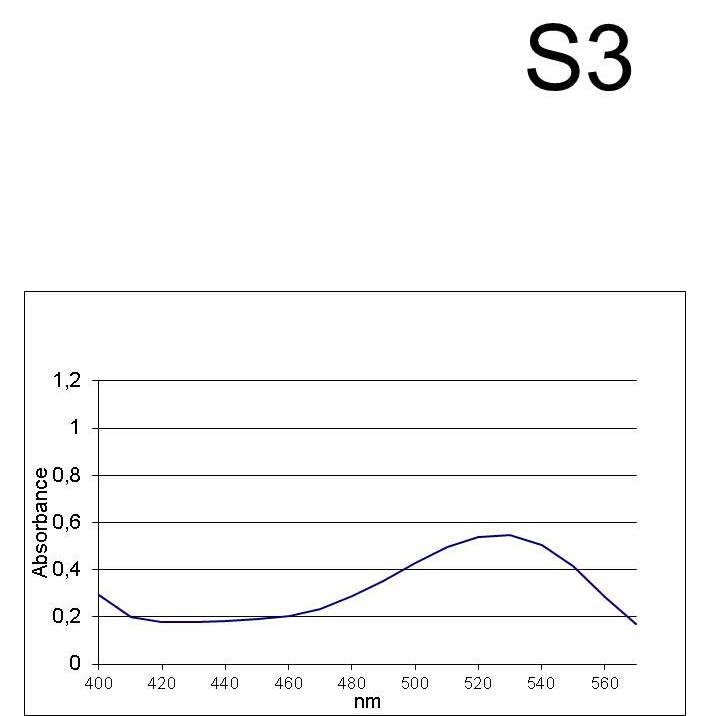

Supplement: Additional file 3 — Figure S3. Absorbance profile of red Poinsettia protoplasts. Anthocyans were extracted by lysis of Poinsettia protoplasts in acidified methanol. Absorbance was assessed using the “absorbance scan” tool of a Tecan microtitre plate reader. [file 1746-4811-8-14-S3.jpeg]

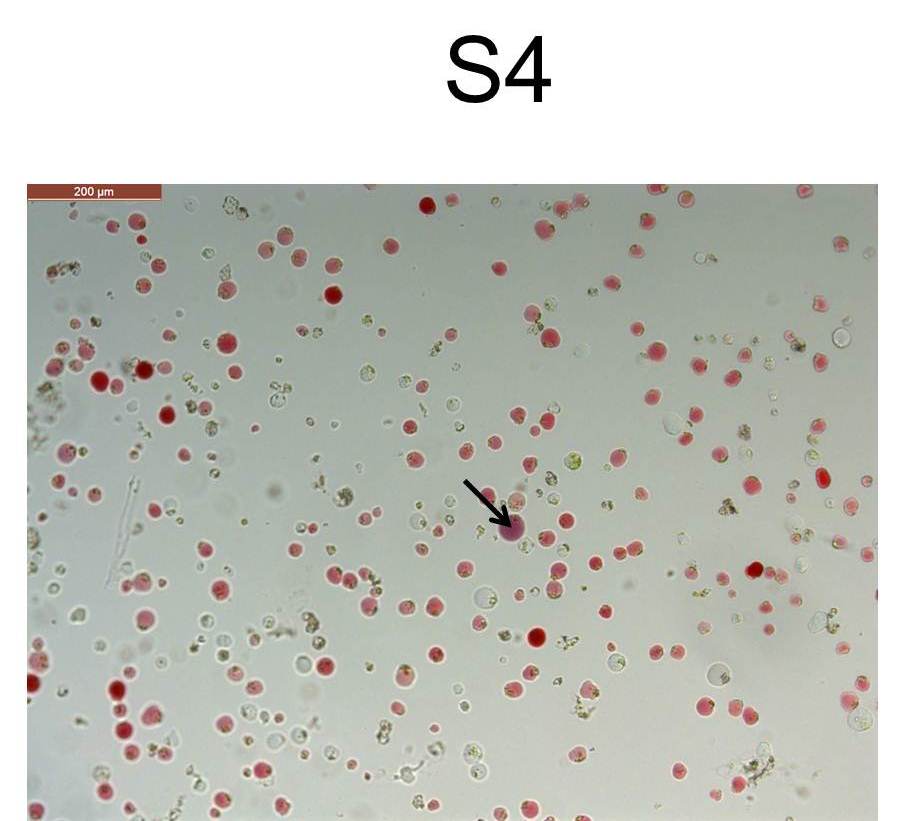

Supplement: Additional file 4 — Figure S4. Viability of Poinsettia protoplasts after the transformation procedure and overnight incubation. Poinsettia protoplasts were incubated for 10 min with Evans blue, a dye which penetrates into non-viable cells. The only non-viable cell contained in this image is indicated by an arrow. [file 1746-4811-8-14-S4.jpeg]

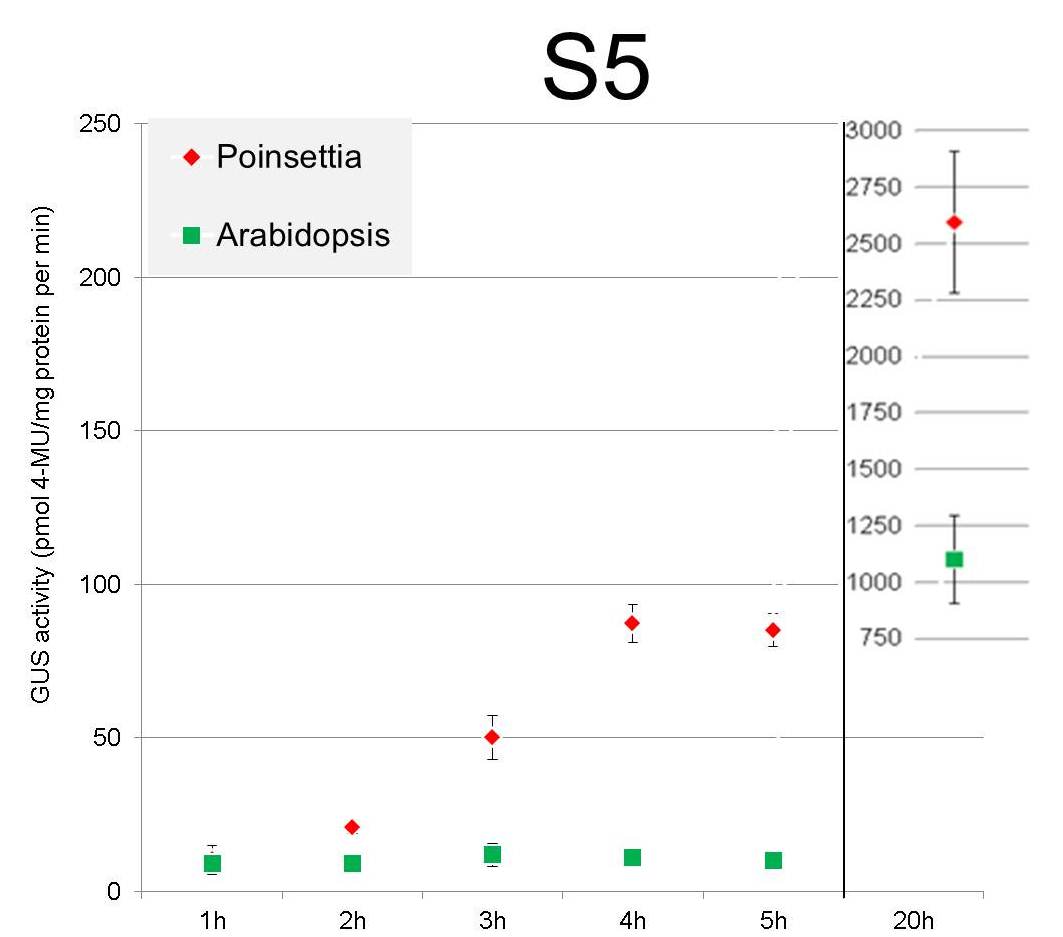

Supplement: Additional file 5 — Figure S5. Kinetics of transgene expression in Poinsettia and Arabidopsis protoplasts.Protoplasts were transfected with 2 μg of a construct for constitutive expression of the glucuronidase (GUS) reporter gene, driven by the CaMV35S promoter. Protoplast samples were collected for GUS activity quantification at the indicated time points post-transformation. [file 1746-4811-8-14-S5.jpeg]

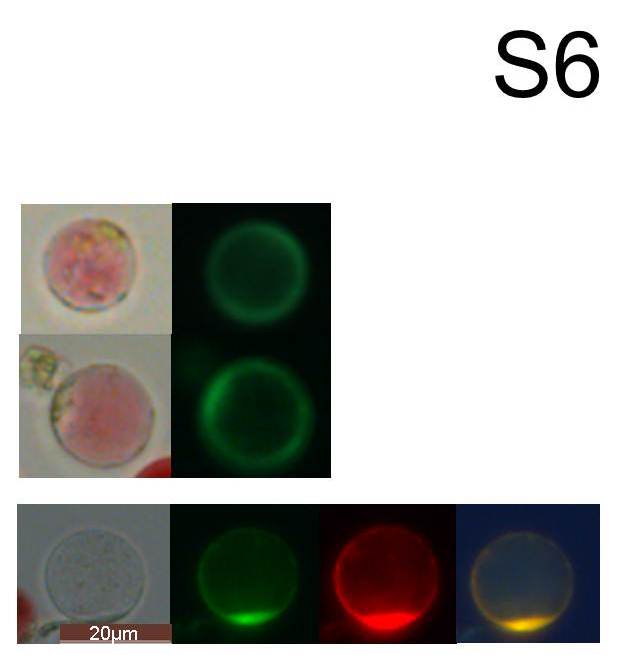

Supplement: Additional file 6 — Figure S6. Subcellular localisation of VIP1-YFP fused to a myristoylation signal peptide. Top: Two examples of Poinsettia protoplasts expressing VIP1-myristoyl-YFP. Bottom: Plasma membrane colocalisation of VIP1-myr-YFP. Poinsettia protoplasts were transformed with VIP1-myr-YFP and treated with the membrane-binding red fluorescent dye FM4-64. Fotographs were taken after 1 hour. From left to right: brightfield, YFP channel, red channel, overlay. Note that chlorophyll–derived autofluorescence is contributing to the extended fluorescent region in the red-channel-image. [file 1746-4811-8-14-S6.jpeg]
